# Anti-Vivisection Ethics

**Published:** 1912-03-23

**Authors:** Beatrice E. Kidd

**Affiliations:** Secretary. British Union for Abolition of Vivisection.


*To the Editor of* The Hospital.

### ANTI-VIVISECTION ETHICS.

*To the Editor of* The Hospital.

Sir,—The "psychology" of anti-vivisectionists, which appears to puzzle the writer of your leading article of March 16, is of a kind which can tackle most problems, but which, I confess, retires confused and nonplussed by the "psychology" underlying the triumphant argument italicised and commented upon in that article. The argument is this : vivisection is alleged to have diminished suffering not only in man, but *in the lower animals*, and it is suggested that this will "be helpful to some who have felt doubts as to man's moral right to help himself to knowledge at the expense of the brute creation!"

Surely it should be unnecessary to point out that anti-vivisectionists would regard the plea for the diminution of human suffering as of more, not less, importance than the diminution of animal suffering! But the point in dispute is not touched by either consideration. That point is whether the individual should be unjustly put to pain for the supposed benefit of others. If the vivisection of individual animals for other animals be a necessity of veterinary science (which we deny), then, logically, the vivisection of human beings becomes a necessity of surgery and medicine, which we deny equally.—Yours, etc.,

British Union for Abolition of Vivisection. Beatrice E. Kidd, Secretary.

